# Optimal sequencing of the first- and second-line target therapies in metastatic renal cell carcinoma: based on nationally representative data analysis from the Korean National Health Insurance System

**DOI:** 10.1186/s12885-023-10991-3

**Published:** 2023-05-30

**Authors:** Dong Hyuk Kang, Joo Yong Lee, Yunhee Lee, U-Syn Ha

**Affiliations:** 1 Department of Urology, Inha University College of Medicine, Incheon, Korea; 2 Department of Urology, Severance Hospital, Urological Science Institute, Yonsei University College of Medicine, Seoul, Korea; 3 Center of Evidence Based Medicine, Institute of Convergence Science, Yonsei University, Seoul, Korea; 4 Department of Urology, Seoul St. Mary’s Hospital, College of Medicine, The Catholic University of Korea, 222, Banpo-Daero, Seocho-Gu, Seoul, 06591 Korea

**Keywords:** Renal cell carcinoma, Sunitinib, Pazopanib, Axitinib, Cabozantinib

## Abstract

**Background:**

The authors intend to compare the effects of each targeted therapy (TT) in the treatment of patients with metastatic renal cell carcinoma (mRCC) using big data based on the Korean National Health Insurance System (NHIS) and determine the optimal treatment sequence.

**Methods:**

Data on the medical use of patients with kidney cancer were obtained from the NHIS database from January 1, 2002, to December 31, 2020. Patient variables included age, sex, income level, place of residence, prescribing department, and duration from diagnosis to the prescription date. The primary outcome was overall survival (OS) for each drug and sequencing. We performed propensity score matching (PSM) according to age, sex, and Charlson Comorbidity Index based on the primary TTs.

**Results:**

After 1:1 PSM, the sunitinib (SUN) (*n* = 1,214) and pazopanib (PAZ) (*n* = 1,214) groups showed a well-matched distribution across the entire cohort. In the primary treatment group, PAZ had lower OS than SUN (HR, 1.167; *p* = 0.0015). In the secondary treatment group, axitinib (AXI) had more favorable OS than cabozantinib (CAB) (HR, 0.735; *p* = 0.0118), and everolimus had more adverse outcomes than CAB (HR, 1.544; *p* < 0.0001). In the first to second TT sequencing, SUN–AXI had the highest OS; however, there was no statistically significant difference when compared with PAZ–AXI, which was the second highest (HR, 0.876; *p* = 0.3312). The 5-year survival rate was calculated in the following order: SUN–AXI (51.44%), PAZ–AXI (47.12%), SUN–CAB (43.59%), and PAZ–CAB (34.28%). When the four sequencing methods were compared, only SUN–AXI versus PAZ–CAB (*p* = 0.003) and PAZ–AXI versus PAZ–CAB (*p* = 0.017) were statistically significant.

**Conclusions:**

In a population-based RWD analysis of Korean patients with mRCC, SUN-AXI sequencing was shown to be the most effective among the first to second TT sequencing methods in treatment, with a relative survival advantage over other sequencing combinations. To further support the results of this study, risk-stratified analysis is needed.

## Background

Kidney cancer is one of the most steadily increasing cancers over the past 30 years, accounting for nearly 74,000 new cases each year in the United States [1]. In Korea, it is the ninth most frequent cancer and eighth most frequent cancer in men [2]. Approximately 20%–30% of patients with newly diagnosed renal cell carcinoma (RCC) have metastases at initial diagnosis, and in the case of local tumors, metastases occur in up to 40% of patients after nephrectomy during follow-up, depending on individual risk factors [3, 4]. Metastatic RCC (mRCC) often has poor prognosis.

Currently, targeted therapies (TTs) and immune checkpoint inhibitors (ICIs) are the main treatments for mRCC. Many TTs have been developed since the mid-2000s, and these have significantly improved the prognosis of both progression-free survival (PFS) and overall survival (OS) in patients with mRCC [5, 6]. Recently, various ICIs other than TTs have been used as new therapeutic agents for mRCC. Numerous studies have demonstrated that ICIs alone or in combination with TTs have better prognosis than conventional TTs alone [7–10]. Therefore, ICI-based monotherapy or combination therapy is currently established as the new first-line treatment for mRCC.

However, in Korea, reimbursement for ICIs was only recently achieved (September 2021), and ICI treatment in patients with mRCC has not been actively implemented in Korea. Therefore, data on this treatment remain insufficient. In addition, many patients have to use only TTs because the use of ICIs is impossible due to their characteristics that can cause serious side effects. For this reason, most patients with mRCC in Korea have been treated by changing TTs for a long period from the mid to late 2000s until recently; it is still considered a major treatment, and reliable data have been accumulated.

As there are various types of TTs for mRCC approved in Korea, optimization of TT sequencing is important. Some studies have been conducted on the optimization of TT sequencing, but they are still insufficient to derive objective and clear results [11–13]. In particular, sunitinib (SUN) and pazopanib (PAZ) are reimbursed as the primary TTs, and axitinib (AXI), cabozantinib (CAB), and everolimus (EVE) are reimbursed as the secondary TTs for patients with mRCC in Korea, and most drugs are used in this order. To date, no studies have compared the effects according to the type and sequencing of the first- and second-line treatments.

Therefore, the authors intend to compare the effects of each TT in the treatment of patients with mRCC using big data based on the Korean National Health Insurance Corporation and determine the optimal treatment sequence.

## Methods

### Database and data collection

Data on the medical use of patients with kidney cancer were obtained from the National Health Insurance System (NHIS) database from January 1, 2002, to December 31, 2020 (NHIS-2022–1-237). The NHIS, launched in 1989, is a universal health insurance system that all Koreans must join and covers approximately 98% of the Korean population [14]. The NHIS manages all medical expenses between individuals, healthcare providers, and the government. Therefore, the NHIS database contains representative and comprehensive information about the medical use of Korean patients, such as patient personal information, demographics, diagnostic codes, prescription drugs and procedures, insurance eligibility, and claim records.

### Study population

**Fig. 1:**
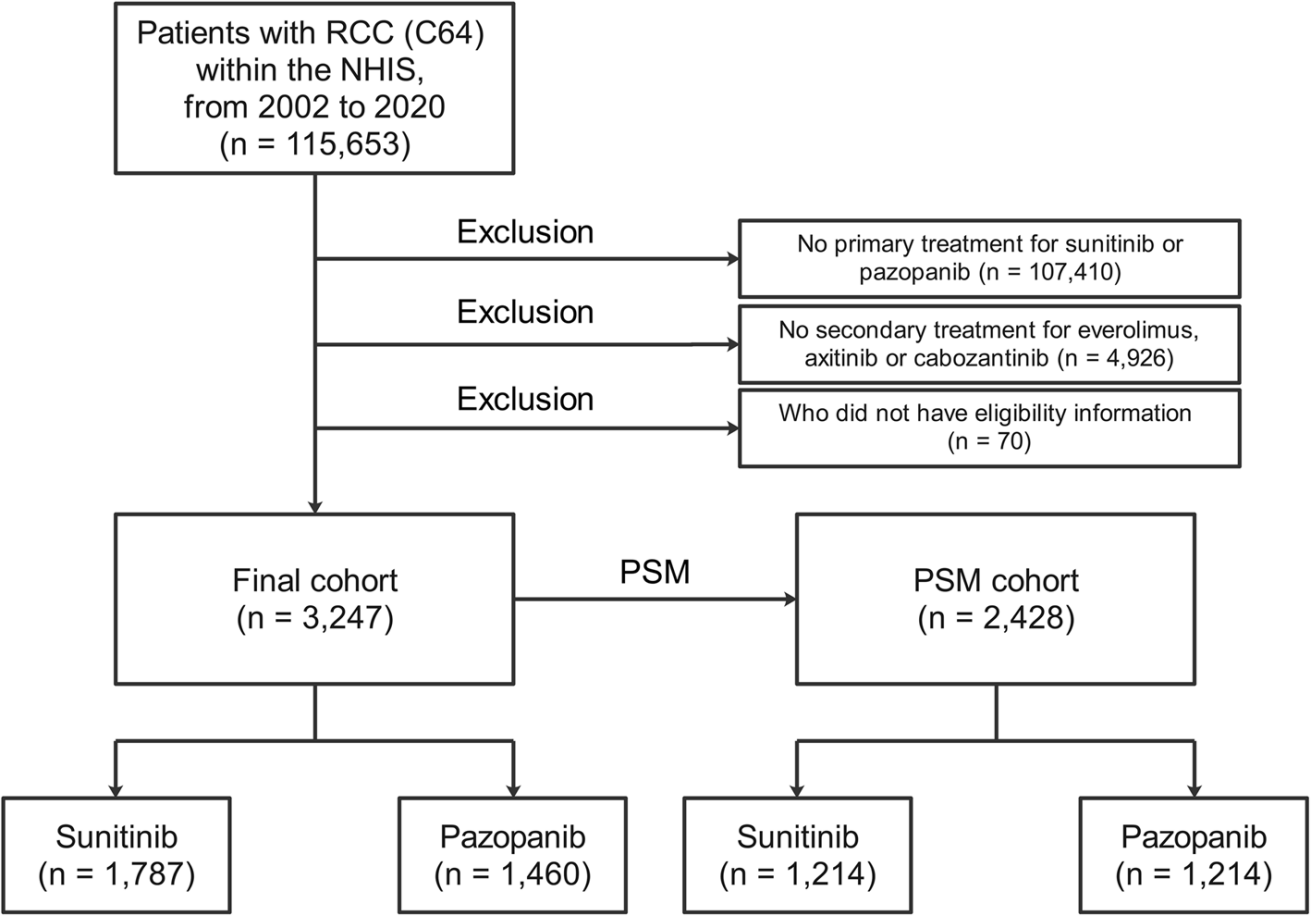
Flowchart showing the participant selection process

Of the 115,653 participants with RCC (diagnostic code, C64) from 2002 to 2020, 8,243 used SUN or PAZ as primary TTs during the same period. Of these, 3,317 patients used EVE, AXI, or CAB as secondary TTs during the same period, of whom 3,247 were included in this study, excluding 70 who did not have eligibility information (e.g., income and place of residence) (Fig. 1).

### Variables

Patient variables included age, sex, income level, place of residence, prescribing department, and duration from diagnosis to the prescription date. Comorbidity status was assessed using the Charlson Comorbidity Index (CCI).

The primary outcome was the OS according to each drug and sequencing, and the secondary outcome compared the OS according to each drug and sequencing by dividing the subgroup from diagnosis to systemic treatment by < 1 year or more.

### Statistical analysis

Patient characteristics were compared based on the first and second TTs. Data are represented as the standard deviation of the mean ± for continuous variables and as a percentage for categorical variables. To compare the mean age according to the first and second TTs, a t-test and ANOVA were performed. Fisher’s exact test and the chi-square test were used to determine the frequency of clinical characteristics. The Cox proportional risk regression analysis was performed on the cohort to generate the risk ratio as a relative risk measure of OS based on the primary and secondary TTs and sequencing. The 5-year survival estimate for OS was calculated using the Kaplan–Meier curve, which was also performed based on the first and second TTs and sequencing. We performed propensity score matching (PSM) according to age, sex, and CCI based on the primary TTs. We performed a 1:1 matching using a caliper set of 0.001. After PSM was performed, the period from the diagnosis date to the systemic treatment was divided into subgroups of < 1 year and more, and further analysis of survival was performed. All statistical analyses were performed using SAS Enterprise Guide 7.1 (SAS Institute Inc., Cary, NC, USA) and R (version 4.2.0; R Foundation for Statistical Computing, Vienna, Australia; http://www.r-project.org). Statistical significance was set at *p* < 0.05.

## Results

### Available TT agents and prescription patterns

**Fig. 2:**
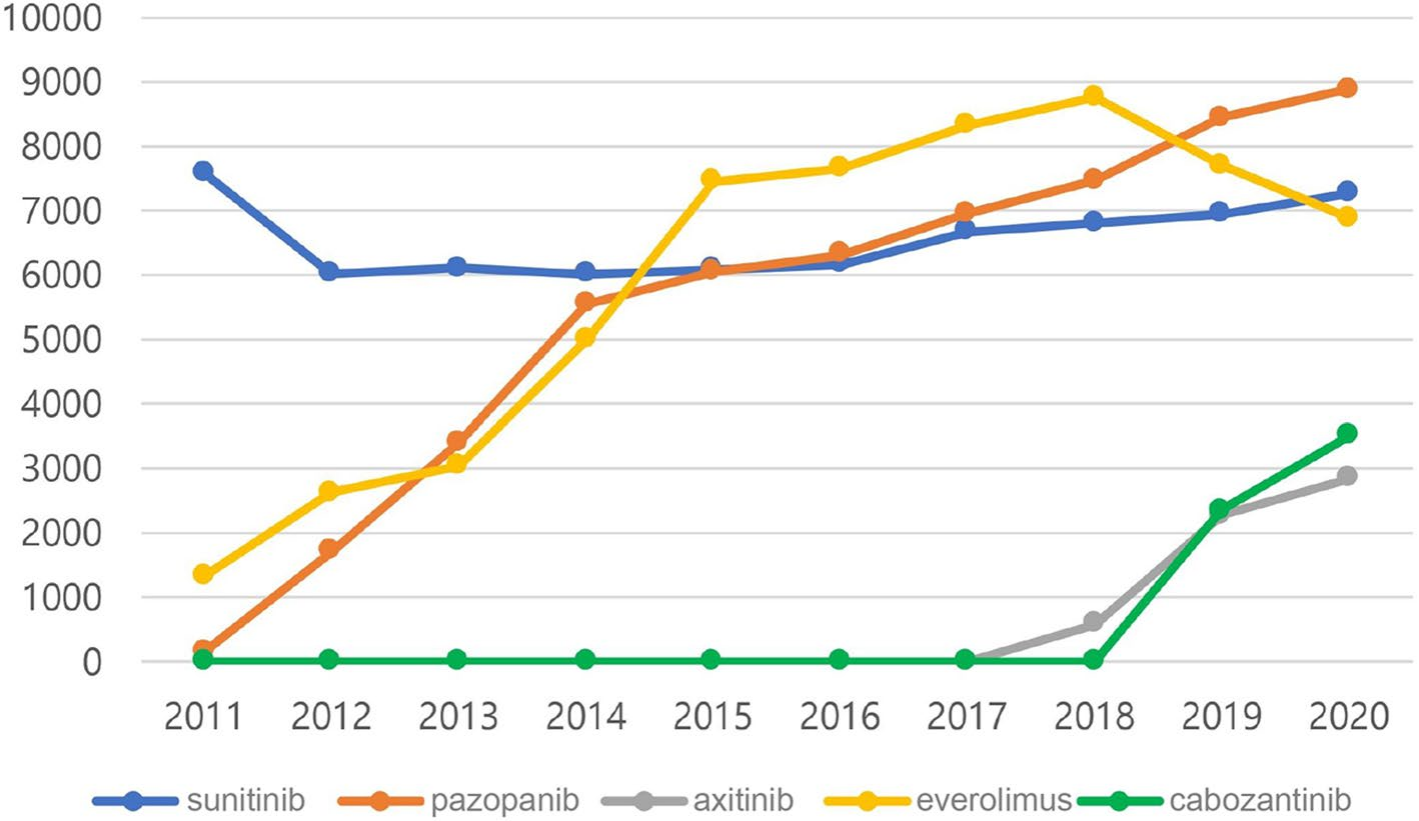
Total number of targeted therapy agent prescription from 2011 until 2020

The TT agents that can be prescribed by national insurance in Korea for the treatment of metastatic kidney cancer are summarized in Table 1. Sorafenib was excluded from the study because of the sharp decline in prescriptions in the country since 2010. Figure 2 shows the number of prescriptions for all TTs prescribed under the Korean health insurance coverage from 2011 to 2020. Since PAZ was approved in 2011, the number of prescriptions has steadily increased every year. Comparing the prescribing pattern with another primary treatment, SUN, the number of prescriptions for SUN exceeded in 2016, and the gap widened every year. The number of EVE prescriptions also increased each year from its approval as a secondary treatment in 2011 until 2018; however, the number of prescriptions has been declining since AXI and CAB were approved as secondary treatments in 2018 and 2019, respectively. Since the approval of AXI and CAB, the number of prescriptions for both drugs has increased.

**Table 1:** Targeted therapy agents that can be prescribed by national insurance in Korea for the treatment of metastatic kidney cancer

Agent	Brand name	National insurance coverage year in Korea	Pathway	Dosing phase	Dosing therapy
Sunitinib	Sutene®	2007	TKI	First or more	P, S
Sorafenib	Nexavar	2007	TKI	First or more	P, S
Pazopanib	Votrient	2011	TKI	First or more	P, S
Everolimus	Afinitor	2011	mTOR inhibitor	Second or more	S
Axitinib	Inlyta	2018	TKI	Second	S
Cabozantinib	Cabometyx	2019	TKI	Second or more	S

*TKI* Tyrosine Kinase Inhibitor, *mTOR* mammalian target of rapamycin, *P* palliative, *S* salvage

### Clinical characteristics of all patients according to the first- and second-line treatments

Of the total 3,247 participants, 1,787 (55.0%) used SUN as the primary TTs and 1,460 (45.0%) used PAZ. In the PAZ group, the mean age was higher than that in the SUN group, and the proportion of women was higher. CCI had a higher rate of intermediate or higher in the PAZ group, and compared with the prescription department, urologists tended to prefer SUN, and internists preferred PAZ. When comparing the secondary drugs, 2,198 patients (67.7%) used EVE, followed by AXI (773 patients, 23.8%) and CAB (276, 8.5%). The mean age was highest in the AXI group, and there was no difference between the three groups in terms of sex and CCI. When compared with prescription departments, internists had a relatively high prescription rate for AXI and CAB compared with urologists. The clinical characteristics of patients treated with primary and secondary drugs for the entire cohort of patients are summarized in Table 2.

**Table 2:** Clinical characteristics according to the first- and second-line treatments

Variables	Total (*n* = 3,247)	First-line treatment			Second-line treatment			
		SUN (1,787, 55.0%)	PAZ (1,460, 45.0%)	p	EVE (2,198, 67.7%)	AXI (773, 23.8%)	CAB (276, 8.5%)	p
Age, mean (SD)	61.27 (10.81)	59.0 (10.05)	64.05 (11.07)	< 0.0001	60.81 (10.77)	62.61 (11.07)	61.16 (10.17)	0.0004
Age group				< 0.0001				0.0106
< 55	849 (26.2)	562 (31.5)	287 (19.7)		610 (27.8)	172 (22.3)	67 (24.3)	
55–59	529 (16.3)	338 (18.9)	191 (13.1)		357 (16.2)	121 (15.7)	51 (18.5)	
60–64	623 (19.2)	380 (21.3)	243 (16.6)		406 (18.5)	153 (19.8)	64 (23.2)	
65–69	459 (14.1)	222 (12.4)	237 (16.2)		316 (14.4)	106 (13.7)	37 (13.4)	
≥ 70	787 (24.2)	285 (15.9)	502 (34.4)		509 (23.1)	221 (28.5)	57 (20.6)	
Sex (%)				< 0.0001				0.2816
Male	2,534 (78.0)	1,444 (80.8)	1,090 (74.7)		1,700 (77.3)	610 (78.9)	224 (81.2)	
Female	713 (22.0)	343 (19.2)	370 (25.3)		498 (22.7)	163 (21.1)	52 (18.8)	
Income level (%)				0.3507				0.3291
0%–20% (lowest)	616 (19.0)	326 (18.2)	290 (19.9)		402 (18.3)	155 (20.0)	59 (21.3)	
20%–40%	468 (14.4)	264 (14.8)	204 (14.0)		318 (14.5)	114 (14.8)	36 (13.0)	
40%–60%	550 (16.9)	319 (17.9)	231 (15.8)		380 (17.3)	121 (15.7)	49 (17.8)	
60%–80%	684 (21.1)	381 (21.3)	303 (20.8)		451 (20.5)	182 (23.5)	51 (18.5)	
80%–100%	929 (28.6)	497 (27.8)	432 (29.5)		647 (29.4)	201 (26.0)	81 (29.4)	
Residence (%)				0.1278				0.02
Metropolitan	1,538 (47.4)	868 (48.6)	670 (45.9)		1,066 (48.5)	333 (43.1)	139 (50.4)	
Other region	1,709 (52.6)	919 (51.4)	790 (54.1)		1,132 (51.5)	440 (56.9)	137 (49.6)	
CCI (%)				0.0071				0.7865
0	1,056 (32.5)	621 (34.8)	435 (29.8)		730 (33.2)	237 (30.7)	89 (32.3)	
1–2	1,643 (50.6)	884 (49.4)	759 (52.0)		1,100 (50.1)	403 (52.1)	140 (50.7)	
≥ 3	548 (16.9)	282 (15.8)	266 (18.2)		368 (16.7)	133 (17.2)	47 (17.0)	
Department (%)								0.0016
Urology	1,445 (44.5)	941 (52.6)	504 (34.5)	< 0.0001	1,021 (46.5)	323 (41.8)	101 (36.6)	
Internal	1,753 (54.0)	825 (46.2)	928 (63.6)		1,145 (52.0)	434 (56.1)	174 (63.0)	
Others	49 (1.5)	21 (1.2)	28 (1.9)		32 (1.5)	16 (2.1)	1 (0.4)	
Time from initial diagnosis to systemic treatment				0.8036				0.1589
< 1 year	1,965 (60.5)	1,078 (60.3)	887 (60.7)		1,355 (61.6)	448 (58.0)	162 (58.7)	
≥ 1 year	1,282 (39.5)	709 (39.7)	573 (39.3)		843 (38.4)	325 (42.0)	114 (41.3)	

*SUN* sunitinib, *PAZ* pazopanib, *EVE* everolimus, *AXI* axitinib, *CAB* cabozantinib, *CCI* Charlson Comorbidity Index

### Survival outcome of all patients according to the first- and second-line treatments and sequencing

The median follow-up period for all patients was 25 months, and the median follow-up and OS results for each drug and sequencing are summarized in Table 3. In the primary treatment group, PAZ had a lower OS than SUN (HR, 1.209; *p* < 0.0001). In the secondary treatment group, AXI was more favorable for survival than CAB (HR, 0.807; *p* < 0.0495) and EVE had more adverse outcomes for survival than CAB (HR, 1.658; *p* < 0.0001). In the first to second TT sequencing, SUN–AXI had the highest OS; however, there was no statistically significant difference when compared with the SUN–CAB combination, which was the second highest (HR, 0.795; *p* = 0.1773) (Table 3).

**Table 3:** Survival outcome according to the first- and second-line treatments and sequencing

Variables	Number (%)	HR	95% CI	p	Median follow-up month (IQR)
Overall survival: first-line treatment					
SUN	1,787 (55.0)	Ref			28
PAZ	1,460 (45.0)	1.209	1.113–1.315	< 0.0001	21
Overall survival: second-line treatment					
CAB	276 (8.5)	Ref			21
AXI	773 (23.8)	0.807	0.652–1.0	0.0495	24
EVE	2,198 (67.7)	1.658	1.377–1.997	< 0.0001	26
Overall survival: sequencing					
PAZ–AXI	473 (14.6)	1.088	0.798–1.484	0.5946	22
PAZ–CAB	152 (4.7)	1.383	0.960–1.991	0.0815	19.5
PAZ–EVE	835 (25.7)	2.423	1.823–3.219	< 0.0001	21
SUN–AXI	300 (9.2)	0.795	0.569–1.110	0.1773	26
SUN–CAB	124 (3.8)	Ref			23
SUN–EVE	1,363 (42.0)	1.760	1.330–2.329	< 0.0001	30

*SUN* sunitinib, *PAZ* pazopanib, *CAB* cabozantinib, *AXI* axitinib, *EVE* everolimus

### Clinical characteristics and survival outcomes of patients after PSM

After 1:1 PSM, the SUN (*n* = 1,214) and PAZ (*n* = 1,214) groups showed a well-matched distribution across the entire cohort. There were no significant differences in the clinical characteristics of most variables, except for prescription (Table 4). In the survival analysis, the median follow-up was 24 months. The median follow-up and OS results for each drug and sequencing are summarized in Table 5. In the primary treatment group, PAZ had a lower OS than SUN (HR, 1.167; *p* = 0.0015). In the secondary treatment group, AXI was more favorable for survival than CAB (HR, 0.735; *p* = 0.0118), and EVE had more adverse outcomes for survival than CAB (HR, 1.544; *p* < 0.0001). In the first to second TT sequencing, SUN–AXI had the highest OS; however, there was no statistically significant difference when compared with the PAZ–AXI combination, which was the second highest (HR, 0.876; *p* = 0.3312).

**Table 4:** Clinical characteristics according to the first- and second-line treatments after age, sex, and CCI matching

Variables	Total (*n* = 2,428)	First-line treatment			Second-line treatment			
		SUN (1,214, 50%)	PAZ (1,214, 50%)	p	EVE (1,614, 66.5%)	AXI (605, 24.9%)	CAB (209, 8.6%)	p
Age, mean (SD)	61.71 (10.52)	61.43 (10.23)	61.99 (10.8)	0.1844	61.58 (10.47)	62.11 (10.88)	61.52 (9.84)	0.5513
Age group				1.0				0.1553
< 55	574 (23.6)	287 (23.6)	287 (23.6)		388 (24.0)	141 (23.3)	45 (21.5)	
55–59	370 (15.3)	185 (15.3)	185 (15.3)		243 (15.1)	91 (15.0)	36 (17.2)	
60–64	484 (19.9)	242 (19.9)	242 (19.9)		302 (18.7)	127 (21.0)	55 (26.3)	
65–69	430 (17.7)	215 (17.7)	215 (17.7)		302 (18.7)	95 (15.7)	33 (15.8)	
≥ 70	570 (23.5)	285 (23.5)	285 (23.5)		379 (23.5)	151 (25.0)	40 (19.2)	
Sex (%)				1.0				0.1443
Male	1,864 (76.8)	932 (76.8)	932 (76.8)		1,220 (75.6)	477 (78.8)	167 (79.9)	
Female	564 (23.2)	282 (23.2)	282 (23.2)		394 (24.4)	128 (21.2)	42 (20.1)	
Income level (%)				0.3779				0.0653
0%–20% (lowest)	485 (20.0)	226 (18.6)	259 (21.3)		307 (19.0)	126 (20.8)	52 (24.9)	
20%–40%	333 (13.7)	161 (13.3)	172 (14.2)		228 (14.2)	83 (13.7)	22 (10.5)	
40%–60%	419 (17.2)	216 (17.8)	203 (16.7)		294 (18.2)	90 (14.9)	35 (16.8)	
60%–80%	529 (21.8)	266 (21.9)	263 (21.7)		338 (20.9)	153 (25.3)	38 (18.2)	
80%–100%	662 (27.3)	345 (28.4)	317 (26.1)		447 (27.7)	153 (25.3)	62 (29.6)	
Residence (%)				0.0959				0.0224
metropolitan	1,173 (48.3)	607 (50)	566 (46.6)		807 (50)	263 (43.5)	103 (49.3)	
Other region	1,255 (51.7)	607 (50)	648 (53.4)		807 (50)	342 (56.5)	106 (50.7)	
CCI (%)				1.0				0.9854
0	786 (32.4)	393 (32.4)	393 (32.4)		528 (32.7)	193 (31.9)	65 (31.1)	
1–2	1,212 (49.9)	606 (49.9)	606 (49.9)		800 (49.6)	306 (50.6)	106 (50.7)	
≥ 3	430 (17.7)	215 (17.7)	215 (17.7)		286 (17.7)	106 (17.5)	38 (18.2)	
Department (%)								
Urology	1,060 (43.6)	650 (53.5)	410 (33.7)	< 0.0001	744 (46.1)	246 (40.7)	70 (33.5)	0.0004
Internal	1,330 (54.8)	551 (45.4)	779 (64.2)		848 (52.5)	344 (56.9)	138 (66.0)	
Others	38 (1.6)	13 (1.1)	25 (2.1)		22 (1.4)	15 (2.4)	1 (0.5)	
Time from initial diagnosis to systemic treatment				0.1467				0.2623
< 1 year	1,463 (60.3)	714 (58.8)	749 (61.7)		990 (61.3)	355 (58.7)	118 (56.5)	
≥ 1 year	965 (39.7)	500 (41.2)	465 (38.3)		624 (38.7)	250 (41.3)	91 (43.5)	

*CCI* Charlson Comorbidity Index, *SUN* sunitinib, *PAZ* pazopanib, *EVE* everolimus, *AXI* axitinib, *CAB* cabozantinib

**Table 5:** Survival outcome according to the first and second-line treatments and sequencing after age, sex, and CCI matching

Variables	Number (%)	HR	95% CI	p	Median follow-up month (IQR)
Overall survival: first-line treatment					
SUN	1,214 (50)	Ref			29
PAZ	1,214 (50)	1.167	1.061–1.283	0.0015	21
Overall survival: second-line treatment					
CAB	209 (8.6)	Ref			19
AXI	605 (24.9)	0.735	0.578–0.934	0.0118	24
EVE	1,614 (66.5)	1.544	1.253–1.903	< 0.0001	25
Overall survival: sequencing					
PAZ–AXI	394 (16.2)	Ref			23
PAZ–CAB	131 (5.4)	1.45	1.073–1.958	0.0155	18
PAZ–EVE	689 (28.4)	2.414	2.015–2.894	< 0.0001	21
SUN–AXI	211 (8.7)	0.876	0.67–1.145	0.3312	26
SUN–CAB	78 (3.2)	1.089	0.749–1.582	0.655	23
SUN–EVE	925 (38.1)	1.769	1.482–2.111	< 0.0001	31

*SUN* sunitinib, *PAZ* pazopanib, *CAB* cabozantinib, *AXI* axitinib, *EVE* everolimus

### Kaplan–Meier survival analysis of patients after PSM

**Fig. 3:**
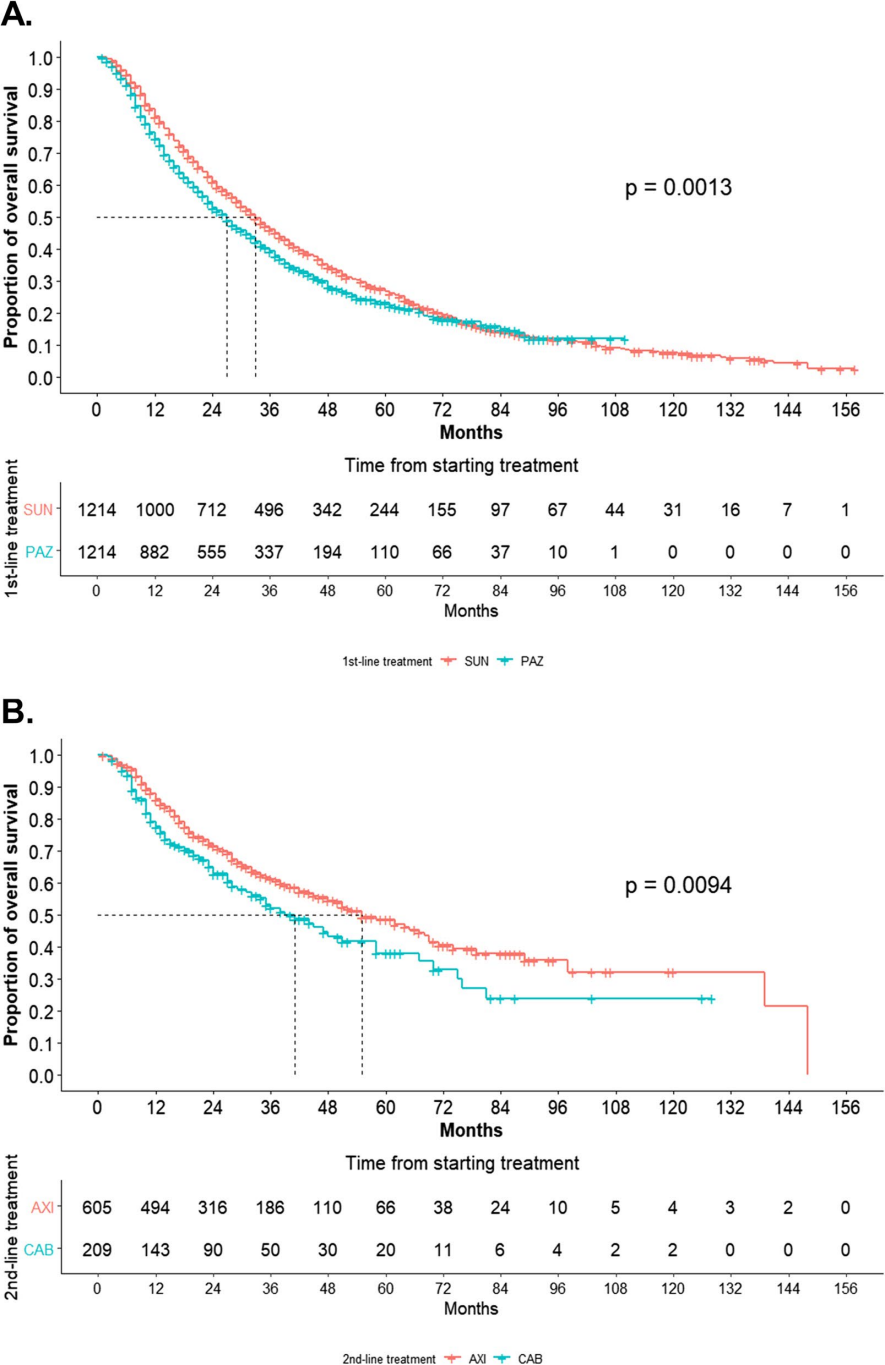
Kaplan–Meier analysis of overall survival stratified by the first- and second-line treatments for patients after age, sex, and CCI matching. **A** SUN versus PAZ and (**B**) AXI versus CAB

**Fig. 4:**
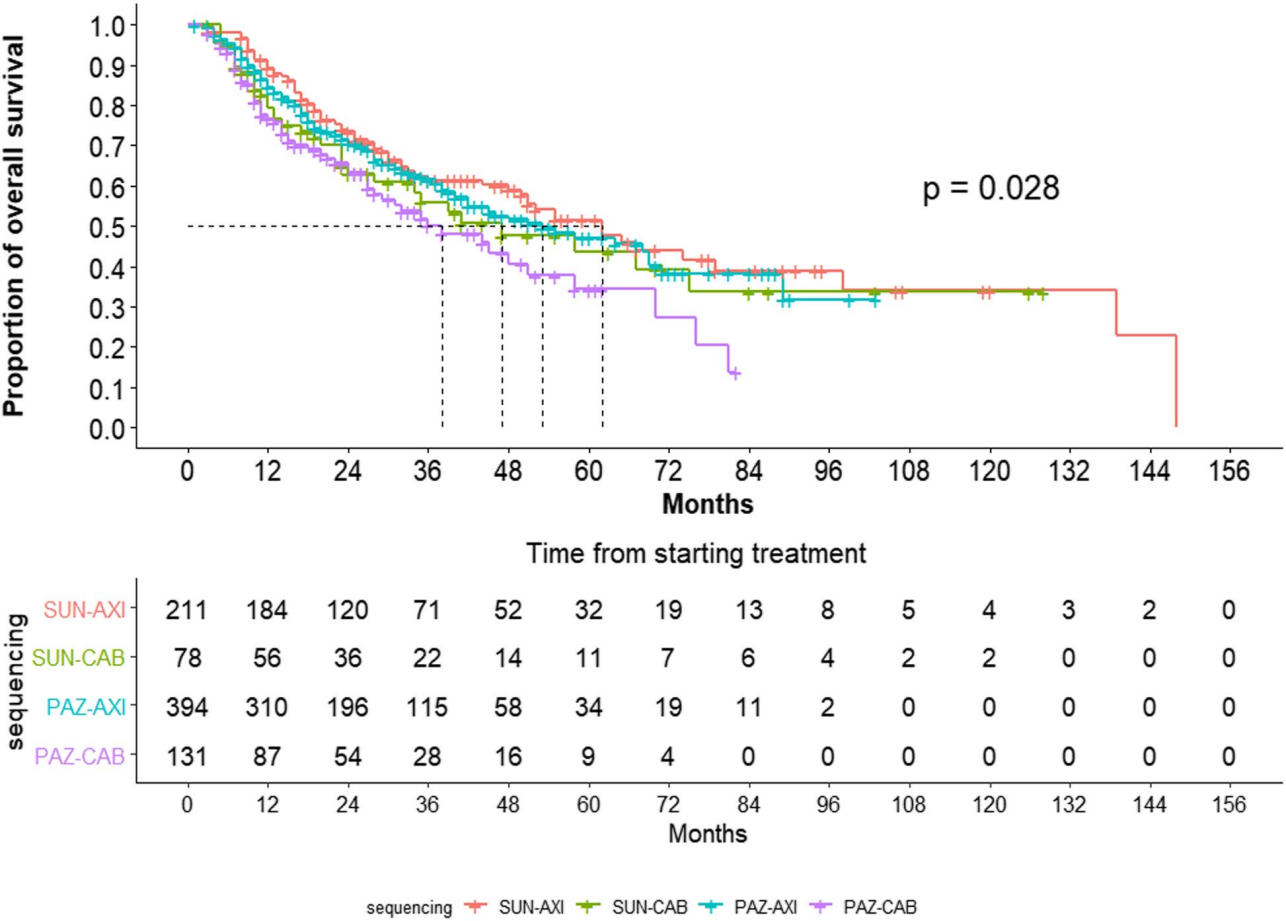
Kaplan–Meier analysis of overall survival stratified by sequencing, except EVE for patients after age, sex, and CCI matching

**Fig. 5:**
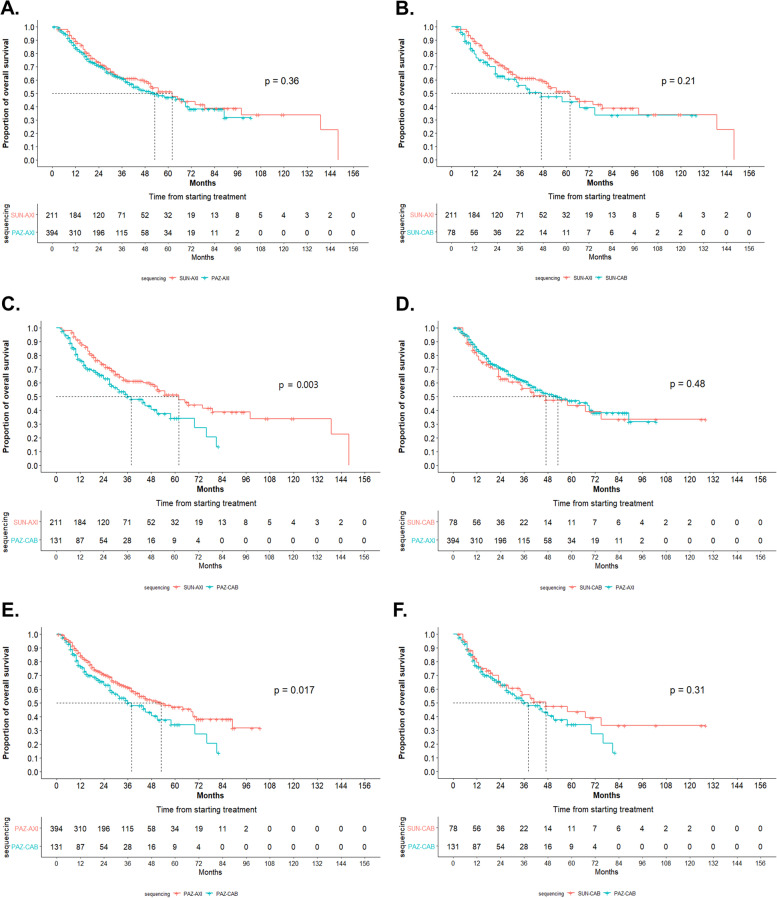
Kaplan–Meier analysis of overall survival stratified by sequencing for patients after age, sex, and CCI matching. **A** SUN–AXI versus PAZ–AXI, **B** SUN–AXI versus SUN–CAB, **C** SUN–AXI versus PAZ–CAB, **D** PAZ–AXI versus SUN–CAB, **E** PAZ–AXI versus PAZ–CAB, and **F** SUN–CAB versus PAZ–CAB

EVE was excluded from the analysis owing to its significantly lower survival outcomes. In the Kaplan–Meier survival analysis, the 5-year survival rate of the primary treatment group with PAZ was lower than that of patients in the SUN group (23.23% vs. 26.89%, *p* = 0.0013) (Fig. 3A). In the secondary treatment group, AXI had a higher 5-year survival rate for CAB (48.75% vs. 38.13%, *p* = 0.0094) (Fig. 3B). In the first to second TT sequencing, the 5-year survival rate was calculated in the following order: SUN–AXI (51.44%), PAZ–AXI (47.12%), SUN–CAB (43.59%), and PAZ–CAB (34.28%) (*p* = 0.028) (Fig. 4). When the four sequencing methods were compared, only SUN–AXI versus PAZ–CAB (*p* = 0.003) and PAZ–AXI versus PAZ–CAB (*p* = 0.017) were statistically significant, and there was no statistical significance in the remaining comparisons (Fig. 5).

### Survival outcome of patients after PSM according to time from initial diagnosis to systemic treatment

When survival analysis was performed on patients whose duration from the first diagnosis to systemic treatment was < 1 year, PAZ still had a lower OS than SUN in the primary treatment group (HR, 1.172; *p* = 0.0081). However, in the second treatment group, there was no statistically significant difference in OS between the AXI and CAB groups (HR, 0.764; *p* = 0.068) (Table 6A). When survival analysis was performed on patients with a period of > 1 year from the first diagnosis to systemic treatment, there was no statistically significant difference in OS between PAZ and SUN in the primary treatment group (HR, 1.12; *p* = 0.1737). In the secondary treatment group, AXI was more favorable for survival than CAB was (HR, 0.61; *p* = 0.0264) (Table 6B).

**Table 6:** Survival outcome of patients < 1 year (A) and > 1 year (B) from diagnosis to systemic treatment according to the first and second-line treatments and sequencing after age, sex, and CCI matching

A. Less than 1 year				
Variables	Number (%)	HR	95% CI	p
Overall survival: first-line treatment				
SUN	714 (48.8)	Ref		
PAZ	749 (51.2)	1.172	1.042–1.319	0.0081
Overall survival: second-line treatment				
CAB	118 (8.1)	Ref		
AXI	355 (24.3)	0.764	0.573–1.020	0.0680
EVE	990 (67.6)	1.337	1.035–1.727	0.0261
Overall survival: sequencing				
PAZ–AXI	226 (15.5)	Ref		
PAZ–CAB	73 (5.0)	1.335	0.929–1.919	0.1187
PAZ–EVE	450 (30.7)	1.873	1.514–2.317	< 0.0001
SUN–AXI	129 (8.8)	0.829	0.609–1.130	0.2352
SUN–CAB	45 (3.1)	1.057	0.674–1.658	0.8099
SUN–EVE	540 (36.9)	1.465	1.189–1.805	0.0003
B. Over than 1 year				
Variables	Number (%)	HR	95% CI	p
Overall survival: first-line treatment				
SUN	500 (51.8)	Ref		
PAZ	465 (48.2)	1.120	0.951–1.317	0.1737
Overall survival: second-line treatment				
CAB	91 (9.4)	Ref		
AXI	250 (25.9)	0.610	0.394–0.944	0.0264
EVE	624 (64.7)	1.841	1.281–2.644	0.0010
Overall survival: sequencing				
PAZ–AXI	168 (17.4)	Ref		
PAZ–CAB	58 (6.0)	1.810	1.053–3.111	0.0318
PAZ–EVE	239 (24.8)	3.577	2.527–5.064	< 0.0001
SUN–AXI	82 (8.5)	0.846	0.492–1.454	0.5446
SUN–CAB	33 (3.4)	1.207	0.616–2.364	0.5835
SUN–EVE	385 (39.9)	2.510	1.791–3.518	< 0.0001

*SUN* sunitinib, *PAZ* pazopanib, *CAB* cabozantinib, *AXI* axitinib, *EVE* everolimus

### Prescription period for each drug

The mean prescribing days for all patients were as follows: SUN, 254.81 ± 267.17; PAZ, 318.09 ± 332.34; EVE, 173.54 ± 243.32; AXI, 175.43 ± 157.71; and CAB, 179.91 ± 161.38 days.

## Discussion

The effects of TTs on patients with mRCC were investigated using nationally representative data from the Korean NHIS. In this study, SUN as a primary agent of TTs in patients with mRCC was more effective in reducing the risk of survival than PAZ, which was more pronounced in the administration group within 1 year. As the secondary TT, AXI is most advantageous in reducing the risk of mortality compared with other drugs, and EVE does not appear to be a reasonable choice for reducing the risk of death. When comparing the sequencing of the first- to second-line treatment after PSM, the SUN–AXI combination was not statistically significant when compared with the PAZ–AXI and SUN–CAB combinations but showed a relatively low HR in survival and a high 5-year survival rate. When compared with the PAZ–CAB combination, the SUN–AXI combination was significantly more effective in terms of survival. Therefore, when the results of this study are taken together, SUN–AXI sequencing is considered to be the most optimal sequencing among the first to second TT sequencing methods when the TT monotherapy is administered in the treatment of mRCC and has a relative survival advantage over other sequencing combinations. In addition, PAZ–AXI and SUN–CAB may be considered alternative sequencing methods; however, PAZ–CAB cannot be recommended without a specific reason.

Many guidelines currently recommend a combination therapy of ICIs + ICIs or ICIs + TTs for mRCC as the first-line systemic treatment [15–17]. Currently, ICIs have recently become widely used in Korea as they have become eligible for insurance benefits. Therefore, since a significant portion of mRCC patients are primarily treated with ICIs, this study may be considered historical rather than informative about patient treatment methods. However, many guidelines recommend TT monotherapy as an alternative treatment when patients are unable to receive or tolerate ICI therapy [15–17]. ICIs have not been eligible for insurance benefits in Korea for a long time, so there is still a lack of accumulated data in actual clinical practice. Additionally, ICIs are not eligible for insurance benefits in favorable risk groups in Korea and can cause serious side effects such as autoimmune diseases, thus TT monotherapy remains a necessary, effective, and important treatment method. Furthermore, there is still a lack of research on TT monotherapy based on population-based real-world data (RWD), particularly on TT monotherapy sequencing. Therefore, we believe that this study will provide important and valid information for actual clinical treatment.

SUN and PAZ are representative anti-VEGF agents and multi-targeted tyrosine kinase inhibitors (TKIs) and are primary TTs licensed as insurance benefits for mRCC treatment in Korea. The comparison of the effects of SUN and PAZ is somewhat controversial; however, through several clinical trials [18–20] and meta-analyses [21], including the COMPARZ study [22], a representative phase 3 randomized controlled trial (RCT), the conclusion that there is no difference between SUN and PAZ is dominant. This differs from the results of our study, in which SUN showed a better effect than PAZ. Several possible reasons for these results can be estimated, and it should be considered that our study had a relatively large number of patients and relatively long follow-up period compared with previous studies. In addition, because our study was not just a comparison of the first drug but also that of a patient who used the second drug, this may have influenced the results. In a real-world study conducted in Canada, OS in SUN showed a significant improvement over PAZ (31.7 vs. 20.6 months, *p* = 0.028) [23]. This is similar to our study in that it used RWD and SUN showed better results. We divided the time from diagnosis to systemic therapy initiation into less than 1 year or later and performed subgroup analysis in the PSM cohort. In the group with less than 1 year from diagnosis and systemic treatment, SUN was shown to be more effective than PAZ. Since this group includes patients with at least one risk factor, they belong to the intermediate to poor risk group. A Canadian study utilizing RWD reported that SUN was superior to PAZ in the intermediate group [23]. Although there was no statistical difference in the poor risk group, SUN showed numerical improvement compared to PAZ. Hence, the results of this Canadian study are consistent with our study findings. Our study demonstrates a larger patient population and longer follow-up duration than previous clinical trials, including COMPARZ. Through our study and other studies in Canada, it suggests that in a population-based real-world clinical setting, RWD analysis may indicate that SUN is more favorable in terms of survival compared to PAZ. Healthcare research using RWD has the advantage that it can reflect the behavior and practices of patients and healthcare providers in the actual clinical field and can optimize the identification of information about effective and economical treatments. Therefore, it is hoped that this will improve the current healthcare and disease management system [24, 25]. We expect that more research using RWD will be conducted on the effects of TTs in mRCC therapy in the future.

The second TTs approved for reimbursement in Korea are AXI and CAB as TKIs and EVE as mTOR inhibitors. To date, many head-to-head trials have not been conducted between these drugs; therefore, there is debate about their effectiveness, and it is difficult to compare their superiority. However, it is clear that EVE was less effective than the other two drugs. The METEOR study [26], a randomized, open-label, phase 3 trial, claimed that the use of CAB was more effective in terms of PFS than EVE. Proskorovsky et al. [27] compared AXI and EVE through an indirect comparison between the METEOR and AXIS studies [28] comparing AXI and sorafenib and found that AXI performed better in OS and PFS than EVE. In addition, in a nationwide database analysis conducted in Hungary, the SUN–AXI sequence had a significantly longer OS than the SUN–EVE sequence therapy [29]. The findings of these previous studies strongly support those of the current research. EVE was found to be significantly less effective than AXI and CAB in all analyses conducted in this study, including overall patients, PSM, and subgroup analyses of < 1 year or more. Domestically, AXI has been available since 2018, and CAB has been available since 2019; therefore, the overall number of prescriptions for secondary drugs is the highest in EVE. However, the impact of these findings is that the number and rate of prescriptions for EVE are believed to be declining significantly, and this decline is expected to intensify in the future. Few studies have compared the effects of AXI with those of CAB. In a base-case analysis conducted by Proskorovsky et al. [27], it was found that there was no difference in OS between AXI and CAB. In our study, AXI showed better results than CAB. More follow-up periods are needed to confirm these results in the future, and well-designed RCT studies are expected to be conducted in the future.

Several studies have been conducted on the sequencing of TTs [11–13]; however, no studies have been conducted on domestically available first- and second-order drugs, namely, SUN–EVE, SUN–AXI, SUN–CAB, PAZ–EVE, PAZ–AXI, and PAZ–CAB sequencing. This study holds significant value as it presents the first analysis of the effect of sequencing using RWD and a simultaneous examination of six different sequencing options. We present SUN–AXI as the optimal sequencing, supporting our findings in the SAX study [30], a real-world study in which AXI was treated sequentially after administration of SUN, with relatively good results at 41.15 months for mOS and 7.14 months for mPFS. Based on our research, further research not only on TTs but also on the sequencing of ICIs or combination therapy is necessary.

This study has several limitations. First, risk assessment is important in the treatment of mRCCs, it was not possible to present criteria based on the International Metastatic Renal Cell Carcinoma Database Consortium (IMDC) or the Memorial Sloan Kettering Cancer Center (MSKCC) model due to the nature of our study using big data collected from the Korean NHIS. To overcome this issue, we divided the time from diagnosis to systemic therapy initiation into less than 1 year or later and analyzed it. In other words, we analyzed one risk factor that can be extracted from our data. Since the less than one year from diagnosis to systemic therapy is common risk factors in both IMDC and MSKCC, indirect adjustment may be possible. There was no significant difference in this factor between each drug group in both the entire cohort and PSM cohort. In addition, because of the nature of the data, side effects could not be evaluated. To address this problem, we compared the mean prescription period for each drug. Considering that SUN administers a 2-week break after 4 weeks of dosing, it is assumed that the total duration of SUN treatment is longer than that of PAZ. This may be because the treatment was more effective, but it may also mean that the patient’s adherence was better. However, this may not be a clear explanation for the side effects, which is a weakness of our study. Between the second drugs, the mean duration of the prescription was approximately the same. Another weakness of our study is the lack of variables such as tumor pathology, burden, or drug dosage. However, since we only focused on stage IV patients managed under strict criteria in the Korean National Health Insurance data, assessing burden was not deemed efficient. Additionally, patients who were not prescribed with approved dosages were excluded from the health insurance coverage and were not analyzed in this study, hence we expect that most patients were administered with typical dosages. Therefore, the possibility of missing variables significantly affecting our study is considered minimal. Finally, one of the regrets is that PFS could not be evaluated. In the entire patient population, there were differences in age, sex, and CCI between the primary drugs; therefore, PSM was conducted, and efforts were made to cancel the variables of dying for reasons other than RCC as much as possible. In addition, in the case of metastatic cancer, it is thought that the OS alone can be representative of the survival effect.

Despite the limitations of this study, it has several valuable features. Firstly, the use of the database of the Korean NHIS, which covers 98% of the population, provides a representative sample of almost all Koreans, making this study a valuable contribution to the field of research. Additionally, the strict criteria applied to the use of TTs for the treatment of mRCC in Korea ensures the reliability of the data. Furthermore, the significant number of patients and long follow-up period further add to the strength of this study. In addition, the comparison and analysis of the results of the six sequencing methods is the first study that has not been conducted until this time, and as the study compared the most sequencing combinations, we believe that the findings of this study have practical implications and can be beneficial for patient care.

## Conclusion

In a population-based RWD analysis in Korea of patients with mRCC, the first agent of TTs was shown to be associated with better survival with SUN compared to PAZ. Among the second agents of TTs, AXI was found to be the most effective in terms of survival, while EVE was not an effective option. Among the first to second TT sequencing methods in the treatment of mRCC, SUN-AXI sequencing was shown to be the most effective, with a relative survival advantage over other sequencing combinations. PAZ-AXI and SUN-CAB may also be considered as alternative sequencing methods, although PAZ-CAB cannot be recommended unless there is a specific reason. To further support the results of this study, risk-stratified analysis is needed. Population-based databases can be expected to guide treatment decisions for physicians and patients in the community setting.

## Data Availability

The datasets generated during and/or analyzed during the current study are not publicly available due to Data Protection Laws and Regulations in Korea, but final analyzing results are available from the corresponding authors on reasonable request.

## Abbreviations

RCC: Renal cell carcinoma
mRCC: Metastatic renal cell carcinoma
TTs: Targeted therapies
ICIs: Immune checkpoint inhibitors
PFS: Progression-free survival
OS: Overall survival
SUN: Sunitinib
PAZ: Pazopanib
AXI: Axitinib
CAB: Cabozantinib
EVE: Everolimus
NHIS: National Health Insurance System
CCI: Charlson Comorbidity Index
PSM: Propensity score matching
TKIs: Tyrosine kinase inhibitors
RWD: Real-world data
RCT: Randomized controlled trial
IMDC: International Metastatic Renal Cell Carcinoma Database Consortium
MSKCC: Memorial Sloan Kettering Cancer Center
